# Development of Chitosan/Mannitol Microparticles as Delivery System for the Oral Administration of a Spirulina Bioactive Peptide Extract

**DOI:** 10.3390/molecules25092086

**Published:** 2020-04-29

**Authors:** Rita P. Aquino, Giulia Auriemma, Giulio M. Conte, Tiziana Esposito, Eduardo Sommella, Pietro Campiglia, Francesca Sansone

**Affiliations:** Department of Pharmacy, University of Salerno, Via Giovanni Paolo II 132, I-84084 Fisciano (SA), Italy; aquinorp@unisa.it (R.P.A.); gauriemma@unisa.it (G.A.); gconte@unisa.it (G.M.C.); tesposito@unisa.it (T.E.); esommella@unisa.it (E.S.); pcampiglia@unisa.it (P.C.)

**Keywords:** *Spirulina* extract, peptides, nutraceutical products, microencapsulation via spray drying, technological properties, stability, in vitro intestinal permeation

## Abstract

*Spirulina platensis* contains several compounds showing nutritional and therapeutic benefits. Recently, a series of peptides able to reduce the blood pressure level and to enhance the endothelial vasorelaxation was isolated from the hydrolyzed highly water-soluble *Spirulina* extract (HSE). However, HSE shows critical organoleptic characteristics also having poor intestinal permeability, limiting absorption when orally delivered. This research aims to overcome the critical issues through the encapsulation of HSE in Chitosan/Mannitol—(CM)-based microparticles by spray drying. The produced powders (CM-HSE) showed good process yield (≈70%) and encapsulation efficiency (≈100%) also having good derived flow properties as well as stability up to six months storage. The microparticles constituting the spray-dried powder resulted in an amorphous micrometric state (d_50_ ≈ 14 µm) able to retain dark colour and unpleasant smell of raw HSE. Moreover, the in vitro permeation study by Franz cell indicated that the engineered microparticles are able to enhance the permeation of HSE through an intestinal biomimetic barrier (551.13 μg/cm^2^ CM-HSE vs. 315.46 μg/cm^2^ HSE at 270 min).

## 1. Introduction

Microalgae of *Arthrospira* species (i.e. *Arthrospira platensis*), known as *Spirulina* have been employed for centuries by several African populations as food, and today are studied for various applications in nutraceutical and food industry [1], thanks to the content of bioactive compounds such as proteins, peptides, carotenoids, chlorophylls, polyphenols, and polyunsaturated fatty acids [2]. Phycocyanin, the main protein of *Spirulina*, has several bioactivities, namely, hepatoprotective, anti-inflammatory, immunomodulating, antioxidant, and anticancer effects [3,4].

Anyway, *Spirulina* biomass and extracts are usually not appreciated by consumers for their organoleptic characteristics and critical sensory attributes (penetrating smell and flavour, dark colour, and undesirable taste). Although sensory attributes do not have a direct relationship with the potential adverse human health effects, they could result in unpalatable products limiting the incorporation in the target food or food supplements. There are many cases reported in the literature that show the difficulty to obtain *Spirulina* bakery products, palatable for the consumers. Shahbazizadeh et al. produced cookies with a supplement of *S. platensis* in a 0.5–1.5 (*w*/*w*) concentration resulting in unpleasant colour, bitter taste and low acceptability [5]; Niccolai et al. developed a microalgae-based bakery product with different concentrations of *Spirulina* biomass (2–6–10% *w*/*w*); whereas the bioactivity was kept with the incorporation of at least 6–10% *w*/*w*, acceptable sensory scores were achieved only up to 2% *w*/*w* of the biomass [6]. Moreover, the bioactivity of its heat-sensitive and low-stability molecules could be compromised during both the storage and heat-treatment manufacturing. 

Another limitation in oral administration of protein-rich extracts is related both to the protein nature itself and their high molecular weight. The proteins in the gastrointestinal environment undergo enzymatic degradations, leading to the production of peptides with a molecular weight distribution of about 1000 Da, which is more easily absorbed [7] and generally responsible for the nutritional value and bioactivity. For this reason, it is very common to subject protein extracts to an in vitro gastrointestinal digestion to obtain a standardized product, less targeted by enzymatic degradations once orally administered [7]. A peptide blend, ideal candidate for the oral intake, should, therefore, have a low molecular weight, high potency, enzymatic/chemical stability as well as high intestinal permeability [8]. Hydrolyzed *Spirulina* extract (HSE) is the digestion product of a *Spirulina* water extract; it is rich in low molecular weight peptides, exerting a significant vasorelaxation activity either on normotensive mice or spontaneously hypertensive rats [9]. HSE contains hundreds of peptides. In detail, 97 peptides, initially encrypted in Phycocyanin and Allophycocyanin (α and β chain), were identified in a mass range from 487.2642 to 2084.0095. As previously reported, to gain insight into the bioactive compounds responsible for this effect, a complex peptidome multistep approach was used to fractionate the crude digest giving five peptide fractions (A–E), of which only fraction E evoked vasorelaxation. The high-resolution mass spectrometry-based screening revealed the presence of four main peptides (spirulina peptides SP3, SP4, SP5, and SP6) in the fraction E, of which only SP6 (GIVAGDVTPI) exerted direct endothelium-dependent vasodilation of ex vivo vessels associated with enhanced serum nitrite levels [9]. Despite the interesting bioactivity, HSE has high water solubility implicating low intestinal absorption due to insufficient epithelial membrane permeability [8,10]. In this case, a formulative strategy to improve permeability, stability, and organoleptic characteristics can be undertaken. 

In the present research, HSE was, for the first time, microencapsulated following a particle engineering approach involving the development of a delivery system based on chitosan/mannitol (CM) matrix able to both enhance the permeation of hydrophilic peptides and convert HSE in a more handling and stable product. CM-HSE was produced by spray-drying with adequate additives providing a physical barrier that protects the core extract from the outside environment reducing its degradation and reactivity [11,12,13]. Additives, such as sugars, polysaccharides, gums, proteins, and lipids may guarantee an easy handling powder with high process yield, low moisture content, and good flowability [14,15]. Mannitol, a stable powdered sugar, was chosen as a loading carrier, as it resists moisture resorption at relatively high humidity and, therefore, it is ideal to load moisture-sensitive extracts [16]. Chitosan was chosen as coating biopolymer, able to promote the formation of microparticles and the efficiency of the spray-drying process [17]; also able to positively affect the membrane permeation as it can open epithelial tight junctions due to its interaction with the negative charges [18], leading to an improvement of the para-cellular transport and increase in the bioavailability of hydrophilic compounds [19]. Due to these characteristics, it has been widely employed for the encapsulation of active ingredients such as peptides and extracts [20,21]. The choice to use a combination of chitosan and mannitol was corroborated by the study of Artursson et al. which demonstrated the mannitol absorption in a Caco-2 monolayers system [22]. Hydrophilic and inert mannitol itself does not distribute in the intestinal mucosa, but in combination with chitosan, an enhancement in permeability was observed [22]. Selecting the matrix and appropriate process conditions, it was possible to obtain engineered particles CM-HSE by spray-drying with interesting characteristics, mainly solid-state, dimensional distribution, and morphology. The final CM-HSE delivery system showed improved technological characteristics, stability, and enhancement of permeation suggesting potential improvement of absorption after oral administration with respect to the unprocessed HSE.

## 2. Results and Discussion 

### 2.1. Microencapsulation Process, Yield, Extract Content, and Encapsulation Efficiency

A challenge in engineered particle design using spray-drying is the balance between optimization of the process parameters and physicochemical characteristics of the material to be encapsulated. An unbalance can result in low product yield due to the low glass transition temperature of feed materials [23] usually found in almost all natural products [24,25]. As a result, the stickiness of the feed materials may form a paste-like soft structure at the wall of the spray dryer and induce poor stability during the storage of the material. The addition of the appropriate additives in right concentrations during the spray-drying process may improve the formation of microparticulate powders and stability [26,27].

A series of pilot experiments were conducted to select the appropriate composition of the feed solution to be spray-dried, in terms of polymer concentration, viscosity, and polymer/extract ratio. Mannitol (M) as a carrier, and chitosan (C) with a low molecular weight (LMW) as coating polymer, were selected in order to have a C concentration high enough to coat extract during the spray drying process but not negatively affecting the shape of microparticles due to the viscosity of the feed [21,28]. Indeed high molecular weight chitosan in elevated concentration, enhancing viscosity of the liquid feed, which is a limiting parameter to spray-drying, can negatively influence the solvent evaporation rate and lower the process yield [21,29].

Therefore, different concentrations of both additives were evaluated; in detail, M was assayed in a range from 1.50 to 2.50% *w*/*v* and chitosan in a range from 0.25 to 1.00% *w*/*v* (data reported in the supplementary file, Table S1). The best results in terms of yield of the process and extract content were obtained employing the highest percentage of M (2.50% *w*/*v*) and the lowest (0.25% *w*/*v*) of C in a ratio of 10:1 (*w*/*w*). This matrix was able to load HSE in a concentration of 0.25% *w*/*v* with a final matrix: extract total ratio of 91.7:8.3).

The obtained results in terms of process yield, extract content, and encapsulation efficiency are reported in Table 1. The content of SP6, the peptide used as a marker [9]*,* in HSE was 1.14% ± 0.23. This value was used as a reference to estimate the HSE content of microparticles.

Process yield for CM-HSE close to 70% was very satisfying considering that a powder recovery greater than 50% is stated to be an index of an efficient spray drying process [14]. Results from UHPLC-MS/MS [9] analysis showed the presence of the SP6 in the same concentration both in HSE raw material and after the microencapsulation process. Thus, the extract content (AEC%) and encapsulation efficiency (EE%) was high, confirming the large number of HSE-loaded particles. Moreover, the loading capacity of the microparticles was calculated [30,31] considering the loss of carrier mass during the process. This value resulted to be about 121 mg of HSE per gram of carrier system indicating a good loading capacity suitable for improved drug dose delivery. 

### 2.2. Powder Characterization

Information on solid-state of the engineered microparticles in terms of morphology and dimensional distribution as well as on the potential extract-polymer interactions and physical stability of materials after spray-drying was derived by Differential Scanning Calorimetry (DSC), morphology and micrometric by Scanning Electronic Microscopy (SEM) and Laser Light scattering (LLS) analysis. Moreover, to evaluate the handling of the powder and the ability to form homogeneous and stable mixtures, powder flowability was assessed and expressed as Hausner ratio and Compressibility Index. The uniformity of content after mixing CM-HSE with other inert excipients was also determined by a powder mixing assay. SEM, LLS, flowability, and hygroscopicity tests were performed immediately and after six months under harsh storage conditions [32]. 

#### 2.2.1. Thermal Analysis (DSC)

**Figure 1:**
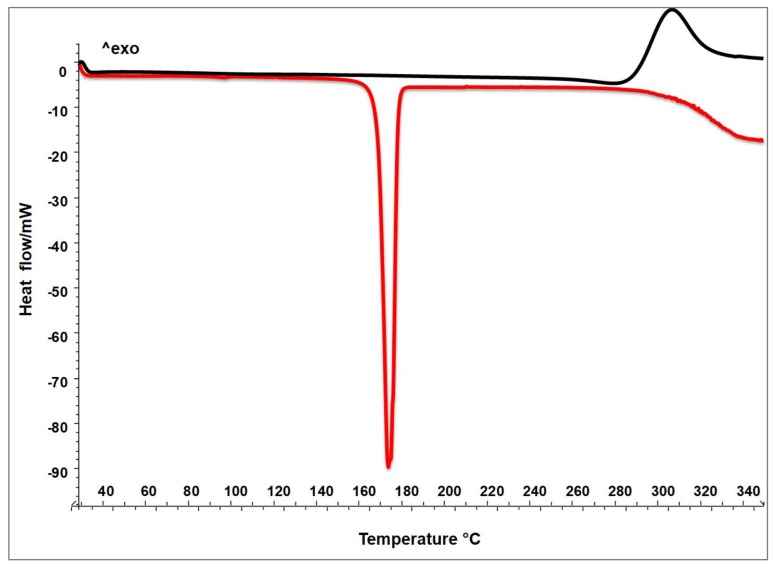
DSC of raw materials: chitosan (C black line) and mannitol (M red line).

The thermograms in Figure 1 show the thermal behaviour of raw mannitol and chitosan. As expected, the thermogram of the crystalline sugar M presents a clear melting onset point at 165 °C and a melting peak at 173 °C (integration between melting onset and endset); that of solid amorphous C shows only an exothermal event around 308 °C due to the crystallization followed by the polysaccharide degradation [21]. 

**Figure 2:**
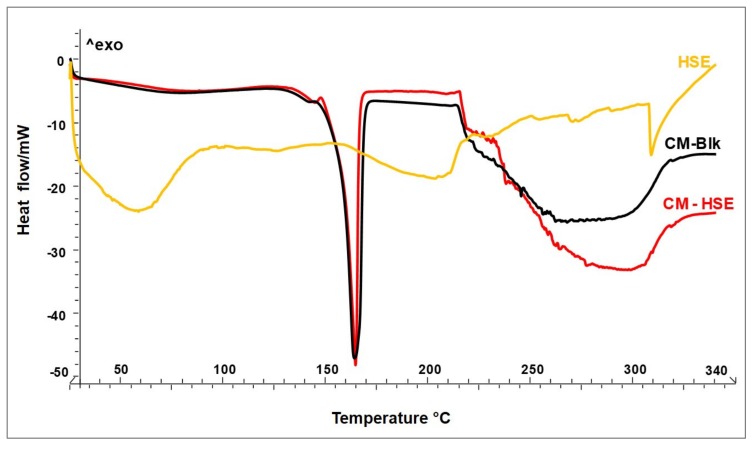
DSC of unloaded CM-Blk (black line) and loaded CM-HSE (red line) particle systems in comparison to HSE raw material (yellow line).

Figure 2 shows the thermograms of unloaded CM-Blk (black line) and loaded CM-HSE (red line) particles systems with respect to HSE raw material (yellow line). The thermal behaviours of both engineered systems (CM-Blk and CM-HSE) highlight the physical interaction between the materials and the amorphization of the final particles. Although the melting point of M is visible at 165 °C in the thermograms of both blank and loaded systems, the first thermal event observable at the baseline is a glass transition at 135 °C typical of amorphous sugar which recrystallizes via glass-transition under the increase in temperature and then melts [29]. 

HSE raw material (yellow line) presents a typical thermogram of natural extracts not showing up a single melting peak, but a series of endothermic events distributed over a wide temperature range from 30 °C (beginning of dehydration, loss of residual humidity) to 230 °C (end of degradation). Within this range, the molecules present in the extract give rise to different endothermic events due to melting or plastic rearrangements [33]. 

A significant result is that HSE degradation begins, as for all natural substances, around 160 °C and ends at 230 °C [28], while the two engineered particle systems begin to degrade after 230 °C. This result could be predictive for greater thermal stability of HSE when embedded in the polymer matrix. No new peaks attributable to chemical interactions were recorded. 

#### 2.2.2. Dimensional Analysis (LLS) at t_0_

The dimensional distribution of particles within the powder system is an important technological parameter influencing many critical properties of particulate materials and is a valuable indicator of quality and performance. As well known, reduced mean size and the shape of particles influence the derived properties of powders, mainly flow, compaction, content uniformity which are of special importance in the manufacturing industry, as well as dissolution rate and consequently in vivo absorption [33].

Raw materials showed high dimensional particle size distribution by LLS analysis (Table 2); d_50_ values ranged from 166.20 µm for chitosan and 125.21 µm for mannitol to 39.76 µm for HSE with a span value of 1.54, 2.47 and 1.89, respectively. A reduced dimensional particle size distribution was recorded for spray-dried systems (d_50_ 4.87 µm for CM-Blk and 14.24 µm for CM-HSE). The higher CM-HSE dimensional distribution with respect to the CM-Blk systems is likely due to the presence of the extract affecting the dimensional distribution and size of particles; the increase in span value (2.66 µm for CM-HSE) with respect to CM-Blk (1.50 µm) is probably ascribable to the presence of small particle aggregates as shown by SEM analysis (see below).

#### 2.2.3. Morphology

The morphological characterizations of the produced systems were conducted by microscopy analysis (fluorescent FM and scanning electron SEM microscopy), and the micrographs were compared to those of HSE raw material. 

**Figure 3:**
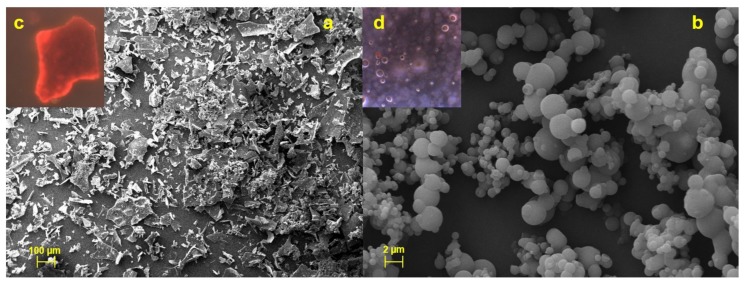
SEM and FM micrographs of HSE (**a** and **c**) and CM-HSE (**b** and **d**).

Unprocessed HSE appears as a crystalline material with irregular shape and surface (Figure 3a) and exploiting red fluorescence (Figure 3c). 

As also reported elsewhere, FM analysis displayed that raw chitosan and mannitol, not being naturally fluorescent substances, emit a blue fluorescence due to the filter used [21] (data reported in Supplementary Materials, Figure S1); CM-HSE microparticles emit a pale yellow fluorescence (Figure 3d) which was the result of the combination between red (HSE) and blue (C and M) fluorescence as evidence of interaction in forming a homogeneous matrix. 

Moreover, SEM analyses indicated that CM-HSE is made by spherical and well-formed microparticles (Figure 3b) obtained during the spray drying process with lower mean particle size (about 2 µm) than LLS analysis (14.24 µm, Table 2). These results suggested the presence of particle aggregates, visible in the picture (3b), resulting in a greater dimensional distribution than actual particle size. Furthermore, the extract is homogeneously distributed and well-encapsulated within the microparticles (Figure 3d). 

Microscopy analyses suggest that the formation of the microparticles was significantly and positively influenced by the balanced mannitol/chitosan ratio; C and M were able to both well interact each other during the spray drying process and embed the extract; moreover, the developed spray-drying conditions led to microsystems consisting of amorphous materials. 

**Figure 4:**
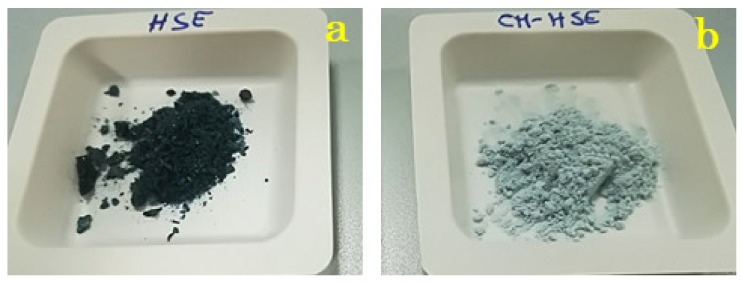
Macroscopic appearance of HSE raw material (**a**) and CM-HSE(**b**).

Amorphous state and encapsulation could have positive effects on flavour retention and colour of the final product. According to literature, the matrix in the amorphous state may be more efficient in aroma retention than crystalline ones which release the encapsulated compounds in a larger amount [34]. Also, additives may improve the colour, depending on the type of feed material and concentration used [35]. Indeed, from an elementary macroscopic point of view, it is possible to notice a change in colour from the dark green of HSE raw material (Figure 4a) to light green of CM-HSE (Figure 4b). 

#### 2.2.4. Derived Powder Properties: Powder Flowability and Mixture Homogeneity

Powder density is an important characteristic for calculating the capacity of packaging, directly correlated to the volumetric distribution. Usually, a powder constituted of particles with heterogeneous shape results in a very low powder flowability due to the poor contact surface area between the large wrinkled and the smaller spherical particles. Thus, a similar powder is not suitable for the filling of a capsule or tablet press [36]. 

To determine the flow properties of the produced systems, Hausner ratio (HR) and Compressibility index (CI) are evaluated (Table 3), based on the tapped and bulk density. An HR below 1.25 with a CI of below 20, according to the U.S Pharmacopeia Scale of flowability, reported in materials and methods section, indicates that powders are free-flowing. 

##### Flowability 

As reported in Table 3 the spray-drying process led to obtaining microparticulate powders CM-HSE with a CI value of 10.67% ± 0.01 and an HR value of 1.12 ± 0.01 starting from an extract HSE with a CI value of 27.67% ± 0.02 and an HR value of 1.38 ± 0.02. The particle engineering approach led to obtaining a powder with enhanced flow properties, compared to the raw HSE extract (Table 3).

##### Mixture Homogeneity

**Figure 5:**
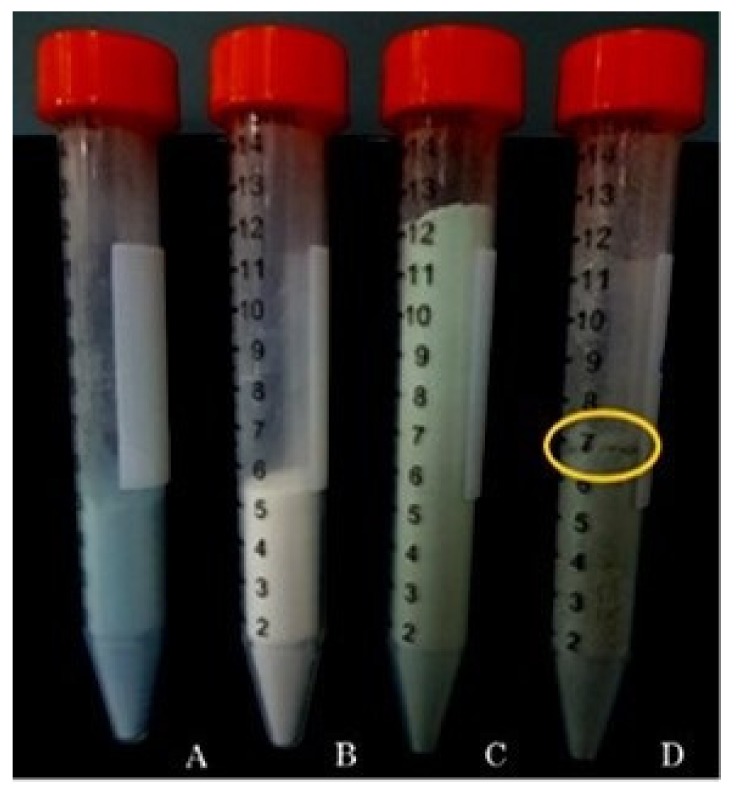
Mixing test on CM-HSE (**A**), Lactose/Mg Stearate 97:3 (*w*/*w*) (**B**), CM-HSE plus Lactose/Mg Stearate 97:3 (*w*/*w*) (**C**) and HSE plus Lactose/Mg Stearate 97:3 (*w*/*w*) (**D**) at T_120_ (after 120 s of mixing).

For the manufacturing industry is important to obtain a handling powder that remains stable and homogeneously mixed during the preparation process of different oral dosage forms, thus suitable for the preparation of supplements in form of pills or tablets [37]. Materials may segregate upon mixing and handling based on differences in particle properties such as size, density, or shape [37]. As shown in Figure 5, samples A, B, and C at the end of the mixing test resulted well packed and sample C constituted of CM-HSE plus Lactose/Mg Stearate resulted in homogeneous mixtures. On the other hand, sample D (HSE plus Lactose/Mg Stearate) resulted partially segregated, with a different breakpoint; this behaviour confirmed the difficulty and the unsuitability of HSE raw material to be incorporated in a solid dosage form.

Solid dosage products with high quality should be designed and manufactured to have a good correlation between the weight (i.e. tablets), mixture homogeneity (i.e. powders) and content uniformity of the produced form. This is done to ensure an accurate and precise dose of the administration [38,39]. For powder forms, the mixture uniformity is correlated to the uniformity of active ingredient content. Uniformity assay showed an HSE content mean value of 14.28 ± 0.83 µg/mg (1.43% *w*/*w*). For each sampling, in each withdrawal point (upper, middle, and lower part of the falcon) of the analyzed mixture, the HSE content was within the limits (75–125% of the mean value) set by the Official Italian Pharmacopoeia [39], so the product complies with the requirements of the test.

### 2.3. Stability Studies

In order to evaluate the shelf-life of the spray-dried systems during the storage period, the solid-state, the flowability powder, and the hygroscopicity were monitored over time [32]. All the samples were tested after six months in harsh storage conditions, both tapped and untapped. Since the obtained results were very similar only the data relative to the tapped samples are shown in the main text (additional data are reported in the supplementary file supplementary file, Tables S2 and S3). 

#### 2.3.1. Dimensional Analysis at t_180_ Days

An increase in shelf-life and maintenance of bioactivity is crucial for the employment of HSE in the food and nutraceutical industry due to the presence of bioactive peptides which are generally known for their fast degradation resulting in loss of biological activity.

**Figure 6:**
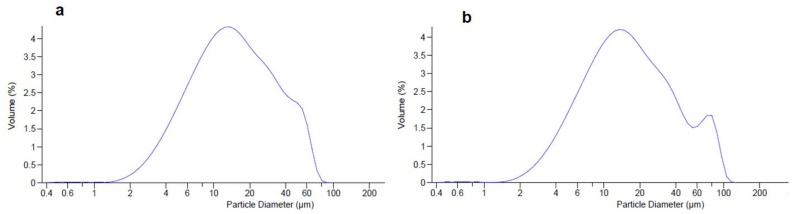
Comparison of dimensional analysis of CM-HSE at t_0_ (**a**) and after six months of storage t_180_ days (**b)**.

HSE, after harsh storage conditions over six months, showed an increase in dimensional distribution (Table 4) with d_50_ of 54.60 µm compared to the starting value of 39.76 µm. This behaviour could be related to the change in the solid-state of the extract from crystalline to final sticky material. As suggested by LLS analysis only a slight increment of particle distribution volume was observed at the end of the storage period) for CM-HSE (Figure 6a,b without significant d_50_ variation from 14.24 µm (a span value 2.66 µm, Table 2) to 15.51 µm (span value 3.21) after six months of storage (Table 4).

#### 2.3.2. Hygroscopicity at t_180_ Days

The hygroscopicity is the ability of a product to absorb or release water vapour from or into the air. Excessive absorption of water by the powder or, conversely, excessive dehydration during storage, leads to changes in the weight of the material which is reflected in the instability during storage. CM-HSE and HSE hygroscopicity were gravimetrically examined after harsh storage conditions to verify the stability over six months. 

No significant changes in the weight (−1.25% ± 0.44) were observed for the microencapsulated powder, indicating that a good encapsulation allowed to have a not hygroscopic final product. For HSE, a weight reduction was observed with a leak in terms of weight of −8.40% ± 1.69 (Table 4).

Altogether the above results suggest that the particle engineering was effective in stabilizing HSE during the microencapsulation process, keeping long-last stability and constant powder properties also under harsh storage conditions.

#### 2.3.3. Morphology at t_180_ Days

**Figure 7:**
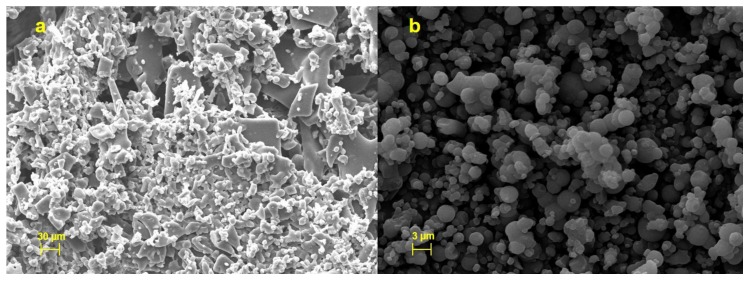
SEM images of HSE (**a**) and CM-HSE (**b**) after 6 months of harsh storage conditions.

Morphology of HSE, at t_180_ days, showed a change in the solid-state from crystalline to sticky material (Figure 7a) and an increase in dimensional distribution as also previously confirmed by LLS analysis. Conversely, CM-HSE resulted unchanged (Figure 7b), after the storage period remained morphologically stable, evidencing particles with a maintained spherical shape, and few aggregation phenomena at t180 days.

#### 2.3.4. Flowability at t180 Days

According to morphological and dimensional analysis, CM-HSE highlighted unchanged flow properties also after the six months storage period (Table 5). Otherwise, it was not possible to evaluate HSE flow properties due to the change in the solid-state well appreciated with SEM micrographs (Figure 7); its stickiness prevented the flow evaluation after six months.

### 2.4. In Vitro Permeability Test

The transport through a simulated intestinal membrane, which can mimic and predict the *in vivo* absorption from the gastrointestinal tract, needs to be evaluated for the engineered particles because of the hydrophilic character of HSE components, mainly peptides. Taking in account the Biopharmaceutics Classification System (BCS), and the two most significant factors influencing oral drug absorption, solubility, and intestinal permeability, HSE may be classified as Class III substance and you can expect low permeation rate through the intestinal membrane in vivo [10]. And with that in mind, CM-HSE was designed as a polymeric matrix including an enhancer of intestinal permeation (chitosan) and a sugar loading carrier (mannitol). 

To study the permeability profile of the produced systems CM-HSE, a biomimetic barrier (Permeapad^®^ Innome GmbH, Germany, UE) able to mimic in vitro the physiological phospholipidic bilayer of the intestinal barrier was used [40]. 

**Figure 8:**
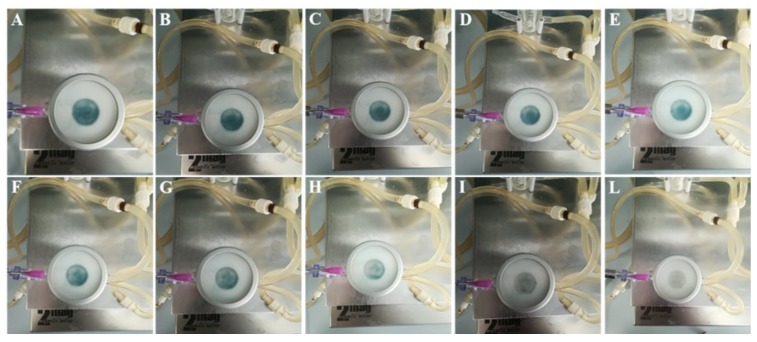
Permeation study–Pictures at different time points, (**A**) 0 min; (**B**) 10 min; (**C**) 20 min; (**D**) 40 min; (**E**) 60 min; (**F**) 90 min; (**G**) 120 min; (**H**) 150 min; (**I**) 210 min; (**L**) 360 min, after CM-HSE application in donor compartment of the Franz cell apparatus.

CM-HSE was gradually absorbed through Permeapad^®^ in the acceptor chamber of Franz Cell apparatus, starting from t_0_ (A) up to 360 min (L), as shown in Figure 8. The typical blue-green colour of CM-HSE, markedly appreciable in the donor compartment of the apparatus in the first pictures (Figure 8A–E), progressively becomes less intense (Figure 8F–I) and completely transparent in L. 

**Figure 9:**
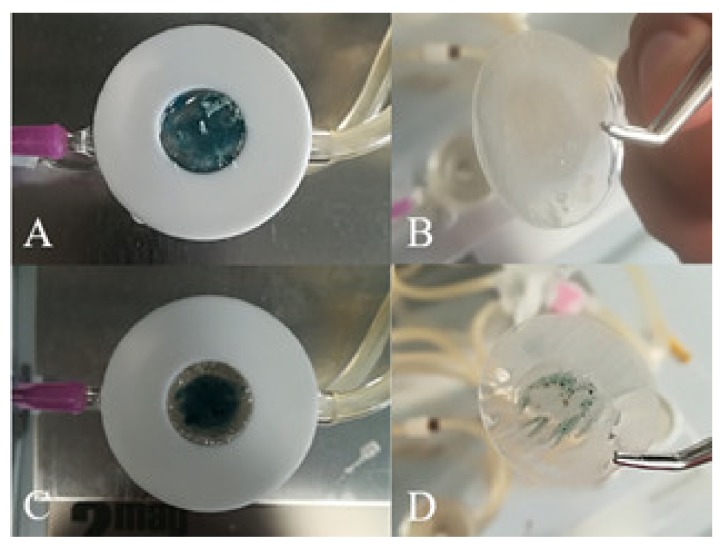
Permeation study—Comparison of Permeapad^®^ membrane loaded with CM-HSE at t_0_ (**A**) and t_360_ (**B**); Comparison of Permeapad^®^ membrane loaded with HSE at t_0_ (**C**) and t_360_ (**D**).

As it is possible to appreciate in Figure 9, while a total permeation of the CM-HSE systems (t_360_, B) through the membrane was observed, HSE (t_360_, D) permeated only partially during the monitored interval of time in the same conditions.

**Figure 10:**
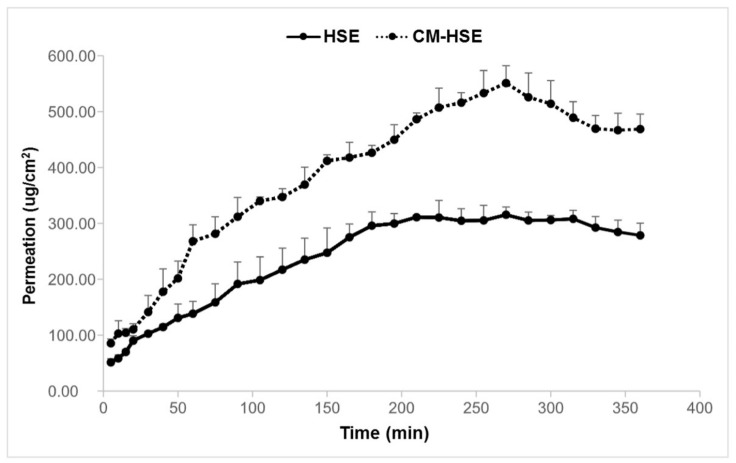
Results of in vitro permeation study of HSE and CM-HSE through Franz Cells.

HSE and CM-HSE permeation were expressed as the amount of permeated extract/permeation area as a function of the time determined by UV analysis and confirmed by UHPLC-MS/MS. As shown in the graph (Figure 10) and according to what is displayed in Figure 10, the amount of HSE permeated through the membrane was higher for the engineered microparticulate system CM-HSE than HSE raw material. The HSE per permeation area, after 270 min, reached the highest value of 551.13 μg/cm^2^, with respect to 315.46 μg/cm^2^ for unprocessed HSE at the same time. 

This study suggests that the enhanced permeation of the extract loaded in the engineered microparticles with respect to raw HSE seems because of the spray-drying process combined with the selected polymer matrix. Especially the ability of chitosan in enhancing the permeation of hydrophilic substances, as reported in the literature [22], is confirmed as well as the correctness of the strategy of a right balance chitosan/mannitol to obtain an improved permeation behaviour of the extract. 

## 3. Materials and Methods

### 3.1. Chemicals

*Spirulina platensis* powder was obtained from FarmaLabor Srl (Canosa di Puglia, Barletta-Trani, Italy).

Ultra-pure water (H_2_O) was obtained by a Direct-8 Milli-Q system (Millipore, Milan, Italy); LC-MS grade acetonitrile (ACN) and water (H_2_O), formic acid (HCOOH), trifluoroacetic acid (TFA), hydrochloric acid, ammonium formate (HCOONH_4_) pepsin, chymotrypsin (bovine pancreas), pancreatin (porcine pancreas), bile salts, Chitosan (C) low molecular weight (LMW ≈150 kDa), degree of deacetylation ≥75% were all purchased from Sigma-Aldrich (St. Louis, MO, USA). Mannitol (M) was purchased from Carlo Erba Reagenti (Rodano, Milano, Italy). Permeapad^®^ barriers were kindly gifted by InnoME GmbH (Espelkamp, Germany). Peptide SP6 (GIVAGDVTPI) was synthesized as reported by Carrizzo et al., 2019 [9], and kindly provided by the authors. 

### 3.2. HSE Preparation

The pre-treatment of 15 g of *Spirulina* powder and aqueous extraction of the protein fraction was carried out following the method reported in our previous work [9]; a recovery over than 50% (7.712 g of lyophilized protein fraction) was obtained. Also the subsequent in vitro gastrointestinal digestion was carried out with the same procedure and apparatus described in reference [8]; the crude GID (gastro-intestinal digestion) hydrolysate was purified by solid-phase extraction (SPE), partially evaporated and then lyophilized giving the final product HSE. 

### 3.3. Spray-Drying Process

#### 3.3.1. Liquid Feed Preparation

The solution of HSE and mannitol was prepared by dissolving 5 g of mannitol in 50 mL of deionized H_2_O adding 0.5 g of HSE and diluting to a volume of 100 mL in a graduated cylinder under magnetic stirring. 

The solution of chitosan was prepared by dissolving 0.5 g of chitosan in 50 mL of acidified water (HCl 0.1 M, pH = 1.27) and diluting to a volume of 100 mL in a graduated cylinder, under magnetic stirring

Both solutions were pooled with a final volume of 200 mL. Each liquid feed was prepared in triplicate.

#### 3.3.2. Spray-Drying Conditions

The feed solution was spray-dried in a mini spray dryer (Büchi B-191-Büchi Laboratoriums-Tecnik, Flawil, Switzerland). The experimental conditions were: inlet temperature of 120 °C, the outlet temperature of 70 °C, air pressure of 6.5 × 10^5^ Pa, drying airflow of 500/600 L/h, aspirator at 90%, spray flow feed rate of 5 mL/min and the nozzle diameter of 0.5 mm. 

Each spray-dried powder was produced in triplicate. All the obtained powders were collected and stored under vacuum for 48 h at room temperature before characterization. 

#### 3.3.3. Yield of Process

The process yield was gravimetrically determined (balance Crystal 100 CAL–Gibertini (max 110 g; d= 0.1 mg; +15 °C/30 °C)) and expressed as the weight percentage of the final product compared to the total amount of the materials sprayed:

(1) $$\mathrm{Yield}\;\%=\left(\frac{\mathrm{weight}\;\mathrm{of}\;\mathrm{final}\;\mathrm{powder}}{\mathrm{weight}\;\mathrm{of}\;\mathrm{all}\;\mathrm{feed}\;\mathrm{component}}\right)\times 100$$

#### 3.3.4. Drug Content

The content of SP6, used as the marker for the characterization of HSE and the engineered particles was estimated by both UV and UHPLC-MS/MS analysis and resulted in 1% (±0.2). 

Briefly, for UHPLC-MS/MS-method analysis were carried out with a Shimadzu Nexera (Shimadzu, Milan, Italy) UHPLC consisting of two LC 30 AD pumps, a SIL 30AC autosampler, a CTO 20AC column oven, a CBM 20A controller, and the system was coupled online to a triple quadrupole LCMS 8050 (Shimadzu, Kyoto, Japan) by an ESI source. The conditions of the separations were: BIOshell TM A160 Peptide C_18_ column with geometry (L × I.D) 100 × 2.1 mm, 2.7 μm (Supelco, Bellefonte, PA, USA) employing as mobile phases: A) 0.1% HCOOH in H_2_O *v*/*v*, B) ACN plus 0.1% HCOOH, with the following gradient starting 0–7 min, 5–40% B; 7–7.01 min, 40–99% B, flow rate set to 0.5 mL/min and the column oven set to 35 °C, with 5 µL of sample injected. Interface temperature, Desolvation line temperature and heat block temperature were set, respectively, to 250 °C, 200 °C and 400 °C. Nebulizing gas, drying (N_2_) and heating gas (air) was set, respectively, to 3, 10 and 10 L/min. 

##### Specificity

To analyze SP6 in raw HSE and CM-HSE, ESI was operated in positive ionization. MS/MS analysis were conducted in multiple reaction monitoring (MRM) (Supplementary Materials, Figure S2), employing as transitions: 941.40 > 731.40 (quantifier ion), Q1 pre-bias −28.0 V, collision energy: −30.0 V, Q3 pre-bias −34.0 V; 941.40 > 612.30 (qualifier ion), Q1 pre-bias −36.0 V, collision energy: −30.0 V, Q3 pre-bias −22.0 V; 941.40 > 343.20, Q1 pre-bias −38.0 V, collision energy: −47.0 V, Q3 pre-bias −23.0 V; Dwell time 50 msec.

For UV-method, a UV double beam spectrophotometer Specord 200 Plus (Analytik Jena, Germany) was equipped with an automatic sampling system using quartz cells (Hellma) with 1 mm pathway. Calibration curves of SP6 (y = 0.001x − 2,1683 r^2^ = 0.9992) and HSE (y = 4090.2x – 2.843 r^2^ = 0.9995) (Supplementary Materials Figures S3 and S4) were previously worked out at 260 nm (λ_max_). The SP6 stock solution was prepared by dissolving 1 mg of the peptide with 1 mL of distilled water. Then, several concentrations in the range of 0.5–500 ng/mL were prepared. The HSE stock solution was prepared by dissolving 1 mg of HSE with 1 mL of distilled water; then several concentrations in the range 5–250 μg/mL. Samples of the particles were prepared by dissolving 5 mg of the spray-dried powders in 5 mL of deionized H_2_O and the absorbance measured spectrophotometrically at λ_max_ 260 nm.

#### 3.3.5. Encapsulation Efficiency 

The extract content, encapsulation efficiency, and loading capacity were calculated by the following equations.

The theoretical extract content (TEC) was calculated as the percentage of the amount of the extract initially loaded in the formulation compared to the amount of all feed components:

(2) $$\mathrm{Theoretical}\;\mathrm{Extract}\;\mathrm{Content}\;\left(\mathrm{TEC}\right)=\left(\frac{\mathrm{weight}\;\mathrm{of}\;\mathrm{HSE}\;\mathrm{fed}\;\mathrm{initially}\;}{\mathrm{weight}\;\mathrm{of}\;\mathrm{all}\;\mathrm{feed}\;\mathrm{components}}\right)\times 100$$

The actual extract content (AEC, amount of HSE in microparticles) was obtained by data interpolation of the powder formulation with calibration curves previously described using SP6 as the marker of HSE (UV method confirmed by UHPLC-MS).

(3) $$\mathrm{Encapsulation}\;\mathrm{efficiency}\;(\%)=\mathrm{AEC}/\mathrm{TEC}=\left(\frac{\mathrm{HSE}\;\mathrm{in}\;\mathrm{microparticles}}{\mathrm{HSE}\;\mathrm{fed}\;\mathrm{initially}\;}\right)\times 100\;$$

The loading capacity was calculated considering the process yield, considering the loss of carrier mass during the process. So the weight (expressed in g) of the carrier in microparticles was calculated as:

Weight of carrier in microparticles = (weight of process yield – weight of HSE in microparticles)(4)

(5) $$\mathrm{Loading}\;\mathrm{capacity}\;(\mathrm{mg}/g)=\left(\frac{\mathrm{weight}\;\mathrm{of}\;\mathrm{HSE}\;\mathrm{in}\;\mathrm{microparticles}}{\mathrm{weight}\;\mathrm{of}\;\mathrm{carrier}\;\mathrm{in}\;\mathrm{microparticles}}\right)\times 1000$$

### 3.4. Powder Characterization—Solid-State Evaluation

#### 3.4.1. Thermal Analysis—Differential Scanning Calorimetry (DSC)

CM-HSE, blank and raw materials (C, M, and HSE) were analyzed by Differential Scanning Calorimetry on an indium calibrated Mettler Toledo DSC 822e (Mettler Toledo, OH, USA). Thermograms were recorded by placing accurately weighed quantities (8–10 mg weighed with a microbalance MTS, Mettler Toledo, OH, USA) of each sample in a 40 µL aluminium pan, which was sealed and pierced. The blank curve was automatically determined by the instrument. The samples underwent one dynamic thermal cycle; they were heated from 25 °C to 350 °C at a heating rate of 10 °C/min.

#### 3.4.2. Dimensional Distribution (LLS)

Sizes and dimensional distributions of CM-HSE and raw materials (C, M, HSE) were carried out with a Laser Light Scattering (LLS) granulometer (Beckman Counter LS 230, Particle Volume Module Plus, Brea, CA, USA).

CM-HSE, blank, and mannitol (15, 20, and 50 mg, respectively) were suspended in 2 mL of ethanol. Approximately 300, 150, and 50 μL, respectively, were added in a small volume cell to obtain an obscuration between 8 and 12%. 

A total of 20 mg of Chitosan was suspended in 2 mL of distilled H_2_O and only a few drops were poured to the same cell obtaining a correct obscuration value.

Particle size distributions were calculated using the Fraunhofer model. The results are expressed as d_10_, d_50_, and d_90_, indicating the volume diameters at the 10th, 50th and 90th percentiles, respectively, of the particle size distribution. The analyses were made in triplicate. The span is defined as:

(6) $$\mathrm{Span}\;\mathrm{value}=\frac{d_{90}-d_{10}}{d_{50}}$$

#### 3.4.3. Morphology (SEM-FM)

The morphologies of raw materials and produced particles were analyzed via scanning electron microscopy (SEM) using a Carl Zeiss EVO MA 10 microscope operating at 17 kV; the powders were coated with Au/Pd and eventually observed at different extensions.

The fluorescent microscopy assays (FM) were performed observing the samples with a Zeiss Axiophot fluorescence microscope, with 40, 63 and 100 × 1.4 NA plan Apochromat oil immersion objectives (Carl Zeiss Vision, München-Hallbergmoos, Germany) using standard DAPI (40, 6-diamidino-2-phenylindole) optics that adsorb violet radiation (max 372 nm) and emit a blue fluorescence (max 456 nm).

#### 3.4.4. Flowability

The bulk and tap densities of CM-HSE was measured as reported in the U.S. Pharmacopeia by a slightly modified method as reported by Sansone et al. [29]. Briefly, CM-HSE was loaded into a bottom-sealed 1 mL plastic syringe (Terumo Europe, Leuven, Belgium) capped with laboratory film (Parafilm1“M”, Pechiney Plastic Packaging, Chicago, IL, USA) and tapped until no change in the volume of the powder was observed. The bulk and the tap densities were calculated, respectively, from the ratio between the net weight of the plastic syringe content and the volume in the syringe before and after tapping. Experiments were performed in triplicate. 

Compressibility index (CI) and Hausner ratio (HR) were calculated using measured values for the bulk and tap densities, as follows:

(7) $$\;\mathrm{Hausner}\;\mathrm{ratio}\;\left(\mathrm{HR}\right)=\frac{\rho_{\mathrm{tap}}}{\rho_{\mathrm{bulk}}}$$

(8) $$\;\mathrm{Compressibility}\;\mathrm{Index}\;\left(\mathrm{CI}\right)=\left(\frac{\rho_{\mathrm{tap}}-{\;\rho }_{\mathrm{bulk}}}{\rho_{\mathrm{tap}}}\right)\times 100$$

The powder flow character was evaluated according to the following classification [29](Table 6).

#### 3.4.5. Mixture Homogeneity

Mixture homogeneity test was performed on CM-HSE, Lactose/Mg Stearate 97:3 (*w*/*w*), CM-HSE plus Lactose/Mg Stearate 97:3 (*w*/*w*) and HSE plus Lactose/Mg Stearate 97:3 (*w*/*w*) (D) after 120 s of mixing.

4 g of Lactose/Mg Stearate (97:3 *w*/*w*) were gently ground for one minute in a mortar with and 2 g of CM-HSE or HSE, then were loaded in a 15 mL falcon, and, finally, mixed with a super mixer for two minutes, to evaluate the resultant mixture homogeneity by macroscopic evaluation.

Uniformity of content was calculated taking three different aliquots from the upper, middle, and lower part of the falcon. Each sample was solubilized in the water a final concentration of 0.500 mg/mL and the HSE content determined spectrophotometrically following the wavelength of 260 nm. 

### 3.5. Stability Studies

Glass vials containing 1 g of CM-HSE was stored for six months at 40 °C ± 2 °C with 75% RH ± 5% (Accelerated stability test, ICH guidelines) [32], in tapped and untapped vials. A climatic chamber was used (Climatic and Thermostatic Chamber, Mod. CCP37, AMT Srl, Milan, Italy).

SEM, LLS, and flowability analyses before storage (t_0_), and for six months (t_180_) were performed.

Hygroscopicity was gravimetrically calculated according to the procedure described by [29,40] and the following formula:

(9) $$\mathrm{Hygroscopicity}\;\left({g\;\mathrm{of}\;H}_{2}O\;\mathrm{per}\;g\;\mathrm{of}\;\mathrm{product}\right)=\frac{\left(m_{180}–m_{0}\right)\;}{m_{180}}\times 100$$

where m_0_ and m_180_ express, respectively, the moisture of the samples before (t_0_) and after (t_180_ days) the storage period. 

### 3.6. In Vitro Permeability Tests

In vitro permeability assay of HSE and CM-HSE was performed using a conventional Franz type vertical diffusion cell (Hanson Research Corporation, Chatsworth, CA, USA), employing Permeapad^®^ (innoME GmbH, Espelkamp, Germany, UE) barrier constituted of Cellulose membrane + Lecithin (S100), able to simulate the passive mass transport through the intestinal membrane [41].

At first, the receptor compartment was filled with 7 mL of PBS (pH = 7.31) and incubated at a temperature of 37 °C ± 0.5 in a water bath. In this compartment, a magnetic stirrer was placed to ensure a constant stirring (250 rpm). The acceptor compartment was, then, covered with Permeapad^®^ barrier, leaving the first hour under stirring to equilibrate the membrane.

Subsequently, the powder (CM-HSE or HSE) was placed on the barrier in the donor compartment until the affected area of mass transfer was totally filled (1.767 cm^2^). The quantity of CM-HSE loaded on the membrane was established in order to fill the surface available for the absorption. As a result, the CM-HSE powder used for the test was of 45 mg, consequently, HSE was loaded with an equivalent amount of 3.8 mg.

The permeation started when 100 μL of PBS was added to the powder and was followed for 6 hours. At time points 5, 10, 15, 20, 30, 40, 50, 60, 75, 90, 105, 120, 135, 150, 165, 180, 195, 210, 225, 240, 255, 270, 285, 300, 315, 330, 345, 360 min, an aliquot of 500 μL was collected from the outlet of acceptor compartment and, simultaneously, replaced from the inlet with fresh PBS at 37 °C. 

Samples collected during the experiment were analyzed spectrophotometrically at a wavelength of 260 nm. Each experiment was performed in triplicate.

An aliquot collected at the end of the equilibration period was used as blank. 

To confirm results by UV analysis, two samples for each hour were also analyzed by UHPLC-MS/MS 

The amount of the extract permeated per area (Q) for each time interval was calculated, according to [42], using the following equation:

(10) $$Q\left(\frac{\mathrm{mg}}{{\mathrm{cm}}^{2}}\right)=\frac{V_{R}{\;x\;C}_{N}+\sum_{i=n}^{n-1}V_{P}{\;x\;C}_{i}\;}{A}$$

where V_R_ is the receiver volume, C_N_ is HSE concentration in the receiver at the time n, V_p_ is the volume of the removed sample, C_i_ is HSE concentration in the receiver at the time n-1. 

Permeation data were reported as the quantity of permeated HSE per permeation area related to time. All the permeation tests were made in triplicate; only the mean values are reported (standard evaluation <1%).

### 3.7. Statistical Analysis

All results are shown as mean ± standard deviation of three experiments performed in triplicate. Statistical comparison between groups was made using ANOVA followed by the Bonferroni parametric test. Differences were considered significant if *p* < 0.05. The SP6 and HSE content data were subjected to one-way analysis of variance (ANOVA) followed by and Tukey HSD test (*p* < 0.05), using GraphPad Prism version 7.00 for Windows.

## 4. Conclusions

A chitosan/mannitol (CM) matrix was studied for its ability to encapsulate via spray drying and to deliver HSE, sensitive peptide-rich *Spirulina* extract. Using a 1:10 C/M ratio, highly efficient encapsulation (100%) of HSE extract was obtained with a good process yield (70%) and production of stable powder made up of well-formed and micronized particles. Bio-active extract HSE was loaded within the microparticles core and enveloped by CM system. Even under harsh storage conditions, neither the dimension, content, morphology, nor derived properties of the particles appeared modified. The spray dried system is potentially able to mask the unpleasant smell and unfavourable taste of HSE and shows a decrease in the initial dark green colour of HSE. Therefore, the engineered particles appear suitable for HSE delivery with good storage, organoleptic, and handling performance. Moreover, the system shows an enhanced permeation of the peptide-rich extract through an in vitro simulating intestinal barrier with respect to the unprocessed HSE. The final product may be proposed as an ingredient for functional food and food supplements for maintaining cardiovascular homeostasis, improving endothelial function, and controlling blood pressure with good technological and manufacturing characteristics. 

The developed method may be generally proposed and applied to carry and deliver extracts with solubility and permeability characteristics like HSE, furnishing a stable product with extended shelf-life and optimized organoleptic, technological, and permeation properties.

## Figures and Tables

**Table 1:** Composition and main characteristics of blank (CM-Blk) and hydrolyzed *Spirulina* extract—(HSE)-loaded particles (CM-HSE).

Samples	M%	C%	HSE%	Yield%	TEC%	SP6%	AEC%	EE%
CM-Blk	2.50	0.25	-	77.03 ± 0.81 ^a^	-	-	-	-
CM-HSE	2.50	0.25	0.25	68.96 ± 0.93 ^a^	8.33	1.17 ± 0.19 ^a^	8.46 ± 0.18 ^a^	101.85 ± 2.05 ^a^

M = Mannitol, C = Chitosan; HSE = Spirulina hydrolyzed extract; TEC = Theoretical Extract Content; AEC = Actual Extract Content; EE = Encapsulation Efficiency (AEC/TEC); ^a^ Average of triplicate analyses ± standard deviation.

**Table 2:** Dimensional distribution (d_50_) of raw materials and spray-dried systems.

Samples	d_50_ μm (Span)
C	166.20 (1.54)
M	125.21 (2.47)
CM-Blk	4.87 (1.50)
HSE	39.76 (1.89)
CM-HSE	14.24 (2.66)

C = Chitosan, M = Mannitol; HSE = Spirulina hydrolysate extract; CM-blk = Blank microparticles; CM-HSE = HSE-loaded microparticles.

**Table 3:** Flow properties of CM-HSE powder.

Samples	Bulk Density (g/cm^3^) ± S.D. *	Tap Density (g/cm^3^) ± S.D.	HR ± S.D.	CI ± S.D.	Flow Character
CM-HSE	369.80 ± 7.16	412.89 ± 9.19	1.12 ± 0.01	10.67% ± 0.01	Good
HSE	140.47 ± 11.01	193.65 ± 14.29	1.38 ± 0.02	27.67% ± 0.02	Poor

* Standard Deviation; the results are expressed as an average of triplicate analyses. Data are mean ± S.D.

**Table 4:** Dimensional distribution (d_50_) of raw materials and spray-dried systems, and hygroscopicity after six months storage period.

Samples	t_180_	t_180_
	d_50_ μm (Span)	Hygroscopicity %
C	/	/
M	/	/
CM-Blk	4.95 (1.28)	/
HSE	54.60 (2.06)	−8.40 ± 1.19
CM-HSE	15.51 (3.21)	−1.25 ± 0.44

C = Chitosan, M = Mannitol; HSE = Spirulina hydrolyzate extract; CM-blk = Blank microparticles; CM-HSE = HSE-loaded microparticles.

**Table 5:** Flow properties of CM-HSE powder after storage (t_180_ days).

Samples	Bulk Density (g/cm^3^) ± S.D. *	Tap Density (g/cm^3^) ± S.D.	HR ± S.D.	CI ± S.D.	Flow Character
CM-HSE (t_180_)	366.11 ± 8.21	411.34 ± 11.12	1.12 ± 0.01	10.33% ± 0.01	Good
HSE (t_180_)	N.D. **	N.D.	N.D.	N.D.	N.D.

* Standard Deviation; ** Not Determined.

**Table 6:** U.S Pharmacopeia Scale of flowability (1174 powder flow)**.**

Compressibility Index	Flow Character	Hausner Ratio
≤10	Excellent	1.00–1.11
11–15	Good	1.12–1.18
16–20	Fair	1.19–1.25
21–25	Passable	1.26–1.34
26–31	Poor	1.35–1.45
32–37	Very poor	1.46–1.59
>38	Very, very poor	>1.60

## Supplementary Materials

The following are available online, Supplementary Material file including Table supplementary 1 (SM1), Table supplementary 2 (SM2), Table supplementary (SM3) and Figure supplementary 1 (SM1), figure supplementary 2 (SM2), figure supplementary 3 (SM3) and figure supplementary 4 (SM4).
