# Supplementary material for: Development of Chitosan/Mannitol Microparticles as Delivery System for the Oral Administration of a Spirulina Bioactive Peptide Extract
Source: Molecules. 2020 Apr 29;25(9):2086. doi: 10.3390/molecules25092086 (PMC7248919; doi:10.3390/molecules25092086)
Supplement: Supplementary file 1 [file molecules-25-02086-s001.pdf]

Article

Development of chitosan/mannitol microparticles as delivery system for the oral administration of a Spirulina bioactive peptide extract

Rita P. Aquino[1], Giulia Auriemma[1], Giulio M. Conte[1], Tiziana Esposito[1], Eduardo Sommella[1], Pietro Campiglia[1] and Francesca Sansone[1*]

[1]Department of Pharmacy, University of Salerno, Via Giovanni Paolo II 132, I-84084 Fisciano (SA), Italy;
* Correspondence: fsansone@unisa.it, Tel.: +39-089968146; Fax: +39-089969602*

Abstract: *Spirulina platensis* contains several compounds showing nutritional and therapeutic benefits. Recently, a series of peptides, able to reduce the blood pressure level and to enhance the endothelial vasorelaxation, were isolated from the hydrolysed highly water-soluble *Spirulina* extract (HSE). However, HSE shows critical organoleptic characteristics also having a poor intestinal permeability limiting absorption when orally delivered. This research aims to overcome the critical issues through the encapsulation of HSE in Chitosan (C)/Mannitol (M) based microparticles by spray drying. The produced powders (CM-HSE) showed good process yield (≈70%) and encapsulation efficiency (≈100%). The microparticles resulted in an amorphous state, possibly able to retain unfavourable odour, colour and taste, with a micrometric dimensional distribution (d_{50} ≈14 μm) also showing good derived flow properties, as well as stability up to six months storage. *In vitro* permeation study by Franz cell indicated how the engineered particles show an enhancement (551.13 *vs* 315.46 μg/cm^2 at 270 min) of permeation through the biomimetic barrier with respect to HSE.

Keywords: *Spirulina* extract; peptides; nutraceutical products; microencapsulation *via* spray drying; technological properties; stability; *in vitro* intestinal permeation.

SUPPLEMENTARY MATERIAL

1. Results

- *Power production*

The obtained results of preliminary experiments to assess the best matrix and process conditions in terms of process yield, SP6 content, extract content and encapsulation efficiency are reported in Table SM1

Table SM1. Composition and main characteristics of produced blanks (CM-Blk) and HSE loaded (CM-HSE) powders

Samples	M %	C %	HSE %	Yield %	SP6 %	AEC %	EE%
HSE	--	--	--	--	1.14±0.23[a]	--	--
C-Blk$_1$	--	1.00	--	28.04±0.37[a]	--	--	--
C-Blk$_2$	--	0.50	--	76.11±0.56[a]	--	--	--
C-Blk$_3$	--	0.25	--	82.04±0.91[a]	--	--	--
CM-Blk$_1$	1.50	0.50	--	38.61±0.64[a]	--	--	--
CM-Blk$_2$	1.50	0.25	--	49.47±1.12[a]	--	--	--
CM-Blk$_3$	2.00	0.50	--	51.64±0.97[a]	--	--	--
CM-Blk$_4$	2.00	0.25	--	62.58±0.84[a]	--	--	--
CM-Blk$_5$	2.50	0.50	--	69.03±0.78[a]	--	--	--
CM-Blk$_6$	2.50	0.25	--	77.03±0.81[a]	--	--	--
CM-HSE$_1$	2.50	0.25	0.15	67.38±1.05[a]	1.19±0.17[a]	4.47±0.16[a]	89.04±2.77[a]
CM-HSE$_2$	2.50	0.25	0.20	68.25±0.77[a]	1.18±0.20[a]	5.96±0.23[a]	99.14±1.85[a]

CM-HSE3	2.50	0.25	0.25	68.96±0.93[a]	1.17±0.19[a]	8.46±0.18[a]	101.85±2.05[a]
CM-HSE4	2.50	0.25	0.30	69.43±0.61[a]	1.08±0.11[a]	7.86±0.14[a]	87.33±1.87[a]
CM-HSE5	2.50	0.25	0.35	66.14 ± 0.58[a]	1.05±0.07[a]	7.48 ± 0.21[a]	68.65 ± 1.62[a]

M = Mannitol, C = Chitosan; HSE = Hydrolyzed Spirulina Extract; AEC= Actual Extract Content; a= Standard deviation

- *Morphology*

Fluorescence Microscopy analysis displayed that raw chitosan and mannitol, not being naturally fluorescent substances, emit a blue fluorescence due to the filter used. Here we show the Blank microparticles CM-BLK6 analysed by FM. As you can see the colour of particles is blue that is due to the DAPI filter used. The fluorescent microscopy assays (FM) were performed observing the samples with a Zeiss Axiophot fluorescence microscope, with 40, 63 and 100 x 1.4 NA plan Apochromat oil immersion objectives (Carl Zeiss Vision, München-Hallbergmoos, Germany) using standard DAPI (40,6-diamidino-2-phenylindole) optics that adsorb violet radiation (max 372 nm) and emit a blue fluorescence (max 456 nm).

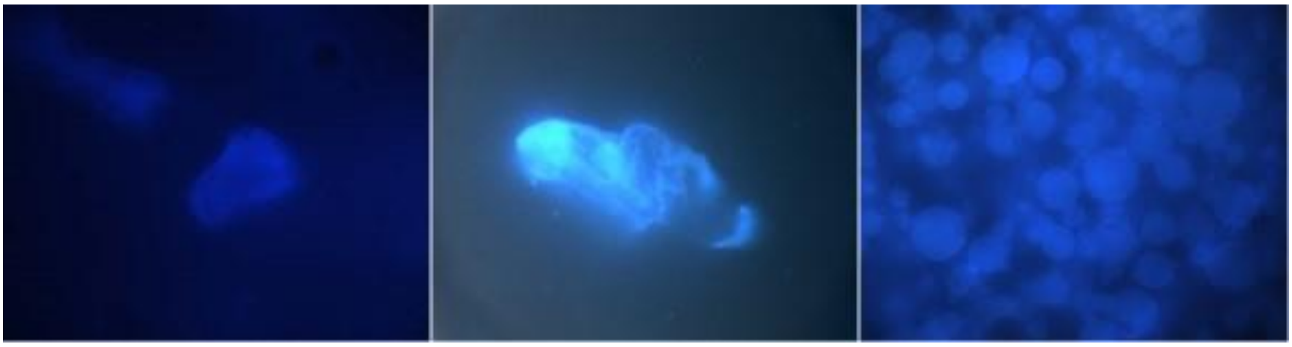

Figure SM1. Blue coloured images of not-naturally fluorescence (starting from the left) mannitol and chitosan raw materials and blank microparticles, influenced by DAPI filter

- *Stability studies*

Glass vials containing 1g of CM-HSE was stored for six months at 40 °C ± 2 °C with 75% RH ± 5% (Accelerated stability test, ICH guidelines), in tapped and untapped vials. A climatic chamber was used (Climatic and Thermostatic Chamber, Mod. CCP37, AMT srl, Milan, Italy). All the samples were tested after six months in harsh storage conditions. All the obtained results are reported in the following tables.

Table SM2. Dimensional distribution (d_{50}) and hygroscopicity at t_0 and after six months in harsh storage conditions in the tapped and untapped system of raw materials and of microparticles

	d_{50} μm (span)		Hygroscopicity
**Samples**	**t_0**	**t_{180}**	**t_{180}**
C	166.20 (1.54)	--	--
M	125.21 (2.47)	--	--
CM-Blk$_{tapped}$	4.87 (1.50)	4.95 (1.28)	--
CM-Blk$_{untapped}$	4.87 (1.50)	4.68 (1.56)	--
CM-HSE$_{tapped}$	14.24 (2.66)	15.32 (3.21)	--
CM-HSE$_{untapped}$	14.24 (2.66)	15.51 (2.61)	-1.25±0.44
HSE$_{tapped}$	39.76 (1.89)	51.57 (2.47)	--
HSE$_{untapped}$	39.76 (1.89)	54.60 (2.06)	-8.40±1.19

Table SM3. Flow properties of HSE and CM-HSE powder tapped and untapped, before and after storage (t_{180} days). The results are expressed as an average of triplicate analyses. Data are mean ± SD.

Samples	Bulk density (g/cm^3) ± S.D.	Tap density (g/cm^3) ± S.D.	HR ± S.D.	CI ± S.D.	Flow character
CM-HSE(t_0)	369.80±7.16	412.89±9.19	1.12±0.01	10.67%±0.01	Good
$CM\text{-}HSE_{tapped}$ (t_{180})	366.11±8.21	411.34±11.12	1.12±0.01	10.33%±0.01	Good
$CM\text{-}HSE_{untapped}$ (t_{180})	368.77±8.21	414.33±6.23	1.13± 0.01	11.00%±0.01	Good
HSE(t_0)	140.47±11.01	193.65±14.29	1.38±0.02	27.67%±0.02	Poor
HSE_{tapped} (t_{180})	N.D.	N.D.	N.D.	N.D.	N.D.
$HSE_{untapped}$ (t_{180})	N.D.	N.D.	N.D.	N.D.	N.D.

- N.D. Not Determined

2. Methods

The content of SP6, used as the marker for the characterization of HSE and the engineered particles was estimated by both UV and UHPLC-MS/MS analysis and resulted of 1% (±0.2). Briefly, for UHPLC-MS/MS-method analysis were carried out with a Shimadzu Nexera (Shimadzu, Milan, Italy) UHPLC consisting of two LC 30 AD pumps, a SIL 30AC autosampler, a CTO 20AC column oven, a CBM 20A controller, and the system was coupled online to a triple quadrupole LCMS 8050 (Shimadzu, Kyoto, Japan) by an ESI source. The conditions of the separations were: BIOshell TM A160 Peptide C18 column with geometry (L × I.D) 100 × 2.1 mm, 2.7 μm (Supelco, Bellefonte PA, USA) employing as mobile phases: A) 0.1 % HCOOH in H2O v/v, B) ACN plus 0.1 % HCOOH, with the following gradient starting 0-7 min, 5-40% B; 7-7.01 min, 40-99% B, flow rate set to 0.5 mL/min and the column oven set to 35 °C, with 5 μL of sample injected. Interface temperature, Desolvation line temperature and Heat Block temperature were set, respectively, to 250 °C, 200 °C and 400 °C. Nebulizing gas, drying (N2) and heating gas (air) was set, respectively, to 3, 10 and 10 L/min. To analyse SP6 in raw HSE and CM-HSE, ESI was operated in positive ionization. MS/MS analysis were conducted in multiple reaction monitoring (MRM) (supplementary material, figure SM2), employing as transitions: 941.40>731.40 (quantifier ion), Q1 pre bias -28.0 V, collision energy: -30.0 V, Q3 pre bias -34.0 V; 941.40>612.30 (qualifier ion), Q1 pre bias -36.0 V, collision energy: -30.0 V, Q3 pre bias -22.0 V; 941.40>343.20, Q1 pre bias -38.0 V, collision energy: -47.0 V, Q3 pre bias -23.0 V; Dwell time 50 msec.

For UV-method, a UV double beam spectrophotometer Specord 200 Plus (Analytik Jena, Germany) was equipped with an automatic sampling system using quartz cells (Hellma) with 1 mm pathway. Calibration curves of SP6 ($y = 0{,}001x - 2{,}1683$ $r^2 = 0{,}9992$) and HSE ($y = 4090{,}2x - 2{,}843$ $r^2 = 0{,}9995$) (supplementary material Figures SM3 and 4) were previously worked out at 260 nm (λmax). The SP6 stock solution was prepared by dissolving 1 mg of the peptide with 1 mL of distilled water. Then, several concentrations in the range of 0.5–500 ng/mL were prepared. The HSE stock solution was prepared by dissolving 1 mg of HSE with 1 mL of distilled water: then several concentrations in the range 5-250 μg/mL. Samples of the particles were prepared by dissolving 5 mg of the spray-dried powders in 5 mL of deionized H_2O and the absorbance measured spectrophotometrically at λ_{max} 260 nm.

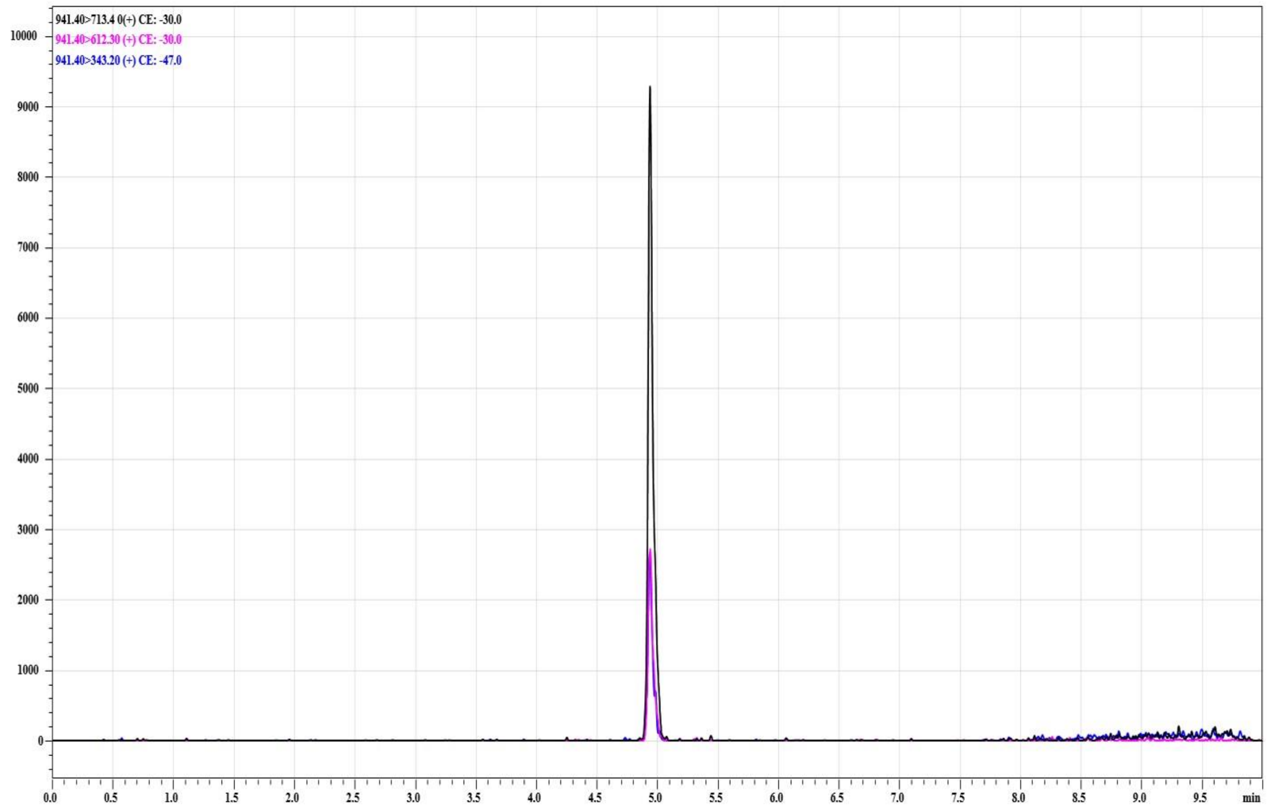

SM2. LC-MS/MS in Multiple Reaction Monitoring of the peptide SP6

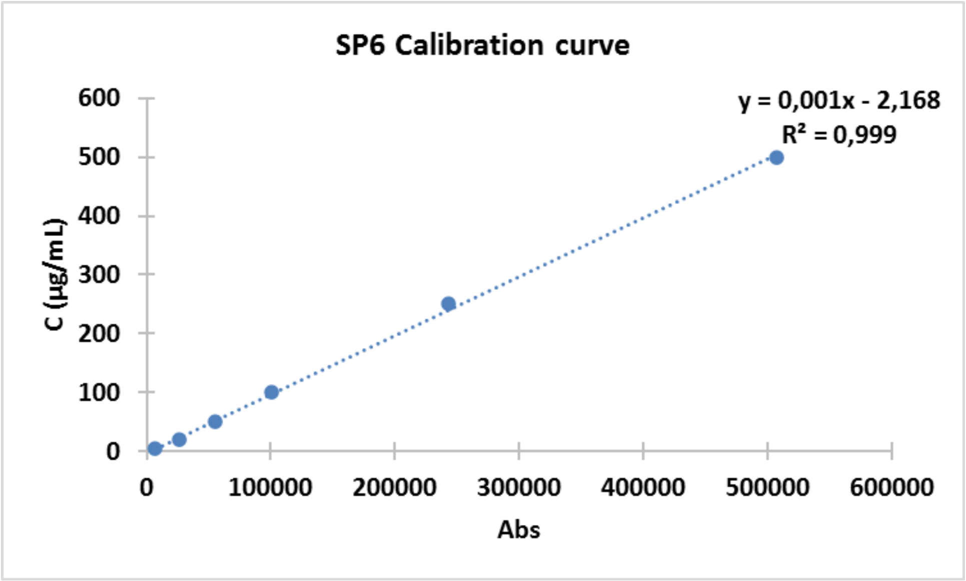

Figure SM3. SP6 calibration curve (LC-MS/MS method)

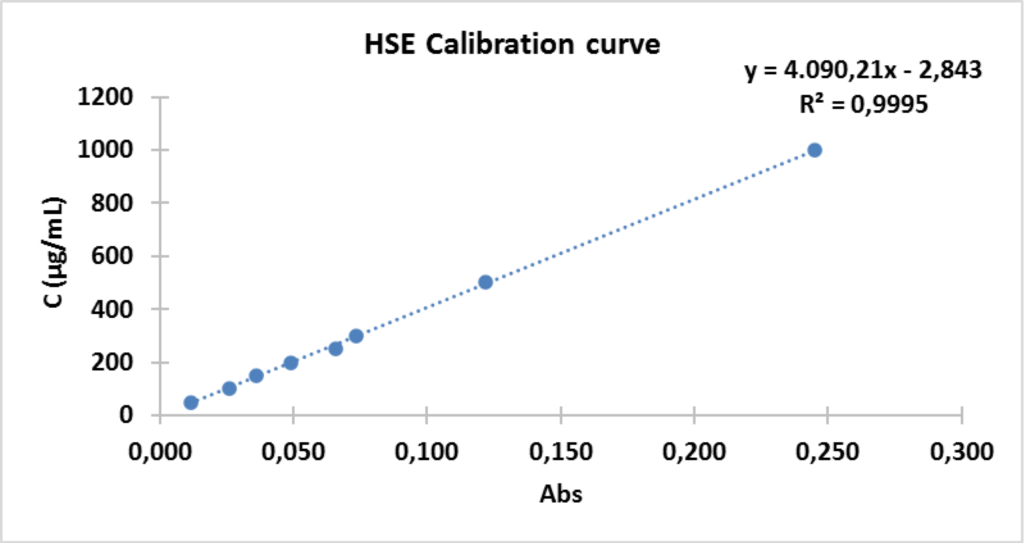

Figure SM4. HSE calibration curve (UV method)
